# Integration of targeted metabolome and transcript profiling of *Pseudomonas syringae*-triggered changes in defence-related phytochemicals in oat plants

**DOI:** 10.1007/s00425-024-04435-w

**Published:** 2024-05-24

**Authors:** Chanel J. Pretorius, Ian A. Dubery

**Affiliations:** Research Centre for Plant Metabolomics, Department of Biochemistry, University of Johannesburg, P.O. Box 524, Auckland Park, Johannesburg, 2006 South Africa

**Keywords:** Avenanthramides, Biotic stress, Host, Metabolomics, Nonhost, Oat, Plant defence, Transcriptomics

## Abstract

**Main conclusion:**

A gene-to-metabolite approach afforded new insights regarding defence mechanisms in oat plants that can be incorporated into plant breeding programmes for the selection of markers and genes related to disease resistance.

**Abstract:**

Monitoring metabolite levels and changes therein can complement and corroborate transcriptome (mRNA) data on plant–pathogen interactions, thus revealing mechanisms involved in pathogen attack and host defence. A multi-omics approach thus adds new layers of information such as identifying metabolites with antimicrobial properties, elucidating metabolomic profiles of infected and non-infected plants, and reveals pathogenic requirements for infection and colonisation. In this study, two oat cultivars (Dunnart and SWK001) were inoculated with *Pseudomonas syringae* pathovars, pathogenic and non-pathogenic on oat. Following inoculation, metabolites were extracted with methanol from leaf tissues at 2, 4 and 6 days post-infection and analysed by multiple reaction monitoring (MRM) on a triple quadrupole mass spectrometer system. Relatedly, mRNA was isolated at the same time points, and the cDNA analysed by quantitative PCR (RT-qPCR) for expression levels of selected gene transcripts associated with avenanthramide (Avn) biosynthesis. The targeted amino acids, hydroxycinnamic acids and Avns were successfully quantified. Distinct cultivar-specific differences in the metabolite responses were observed in response to pathogenic and non-pathogenic strains. Trends in aromatic amino acids and hydroxycinnamic acids seem to indicate stronger activation and flux through these pathways in Dunnart as compared to SWK001. A positive correlation between hydroxycinnamoyl-CoA:hydroxyanthranilate *N*-hydroxycinnamoyl transferase (*HHT*) gene expression and the abundance of Avn A in both cultivars was documented. However, transcript profiling of selected genes involved in Avn synthesis did not reveal a clear pattern to distinguish between the tolerant and susceptible cultivars.

**Graphical abstract:**

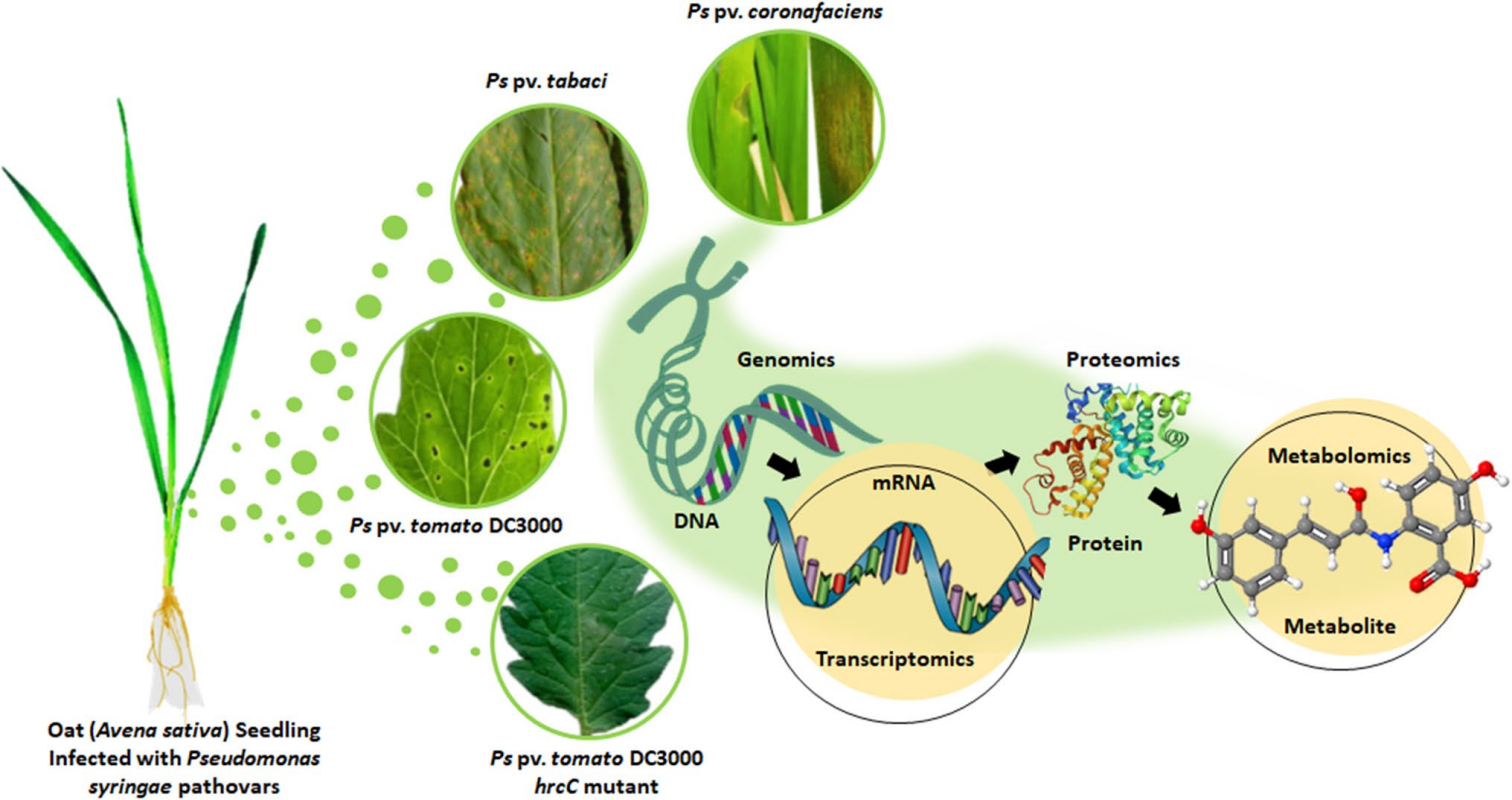

**Supplementary Information:**

The online version contains supplementary material available at 10.1007/s00425-024-04435-w.

## Introduction

As one of two distinct groups of metabolomics methodologies, targeted metabolomics is aimed at the absolute quantitation of known selected or discriminant compounds, as opposed to data-driven untargeted metabolomics, which profiles as many metabolites as possible in a given set of data. Targeted metabolomics, as a hypothesis-driven strategy, mainly emphasises the detection of clearly defined metabolites to obtain a broad understanding of specific pathways. This type of metabolomics is often carried out on a triple quadrupole mass spectrometer, while untargeted metabolomics analyses are performed using high-resolution mass spectrometers for accurate mass identification (Roberts et al. 2012; Schwaiger-Haber et al. 2021). Multiple reaction monitoring (MRM)-based analyses on a triple quadrupole mass spectrometer offer high sensitivity, selectivity, reproducibility, and a broad dynamic range when combined with liquid chromatography-based separation platforms. These characteristics make MRM-based methods ideal for the analysis of multiple analytes in complex biological samples. Targeted metabolomics analyses have a wide range of potential uses, such as early disease detection, biomarker identification, and insight into certain metabolic fluxes and pathways (Shi et al. 2012; Zhou and Yin 2016).

The *Pseudomonas syringae* species complex includes several plant-specialist phylogroups, divided into more than 60 pathovars (Xin et al. 2018). *P. syringae* attacks potential host plants using various virulence factors such as effector proteins to penetrate the host cell membranes through type III secretion systems (T3SS). Also secreted are exopolysaccharides and cell wall-degrading enzymes, as well as small-molecule toxins and plant hormone mimics (Xin and He 2013). This allows successful pathovars the ability to form an aqueous apoplast niche from which innate host immunity can be repressed (Guo et al 2009; Xin et al. 2018).

An untargeted metabolomics approach was previously applied on halo blight susceptible (SWK001) vs tolerant (Dunnart) oat cultivars and revealed pathways altered in response to treatment/infection with *P. syringae* pv. *coronafaciens* (*Ps-c*). Among the reprogrammed pathways, general secondary metabolite biosynthesis and specifically the phenylpropanoid pathway were revealed to play an important role in oat defence (Pretorius et al. 2022). It is well known that phenolics occupy an essential role of chemical defence in the plant immune system and are therefore critical in determining how plants react to diverse disease-causing pathogens. Phenolic compounds (synthesised via the phenylpropanoid pathway) are utilised in the synthesis of avenanthramides (Avns) from phenylalanine, initiated by the stress-responsive enzyme phenylalanine ammonia-lyase (PAL) and the subsequent hydroxylation of cinnamic acid to *p*-coumaric acid by cinnamic acid 4-hydroxylase (C4′H). After being transformed by 4-coumarate-CoA ligase (4CL) into its active CoA thioester analogue, *p*-coumaroyl-CoA (the donor molecule) is conjugated to 5-hydroxyanthranilic acid (the acceptor molecule), catalysed by hydroxycinnamoyl-CoA:hydroxyanthranilate *N*-hydroxycinnamoyl transferase (HHT), to produce Avn A (Pretorius and Dubery 2023, Fig. 1). These enzymes are key in the synthesis of Avns. Six HHTs and one CCoAOMT cDNA have been identified and isolated from hexaploid *Avena sativa*. Among these, *AsHHT1–3* and *AsHHT4–6* isoforms have high levels of amino acid sequence identity: 95–98% and 95%, respectively. On the other hand, it has been found that the *AsHHT4–6* isoforms have relatively lower levels of identity with that of *AsHHT1–3*, only showing 82% similarity (Li et al. 2019; Kim et al. 2021a). According to a study by Yang et al. (2004) on oat leaves inoculated with *Puccinia coronata* f. sp. *avenae*, an increased expression of *AsHHT1* and *AsCCoAoMT* was noted in both compatible (successful pathogen, i.e. disease causing) and incompatible interactions (successful plant, i.e. host defence). This observation pointed to the importance of these phytoalexins in the chemical defences of oat plants.

**Fig. 1:**
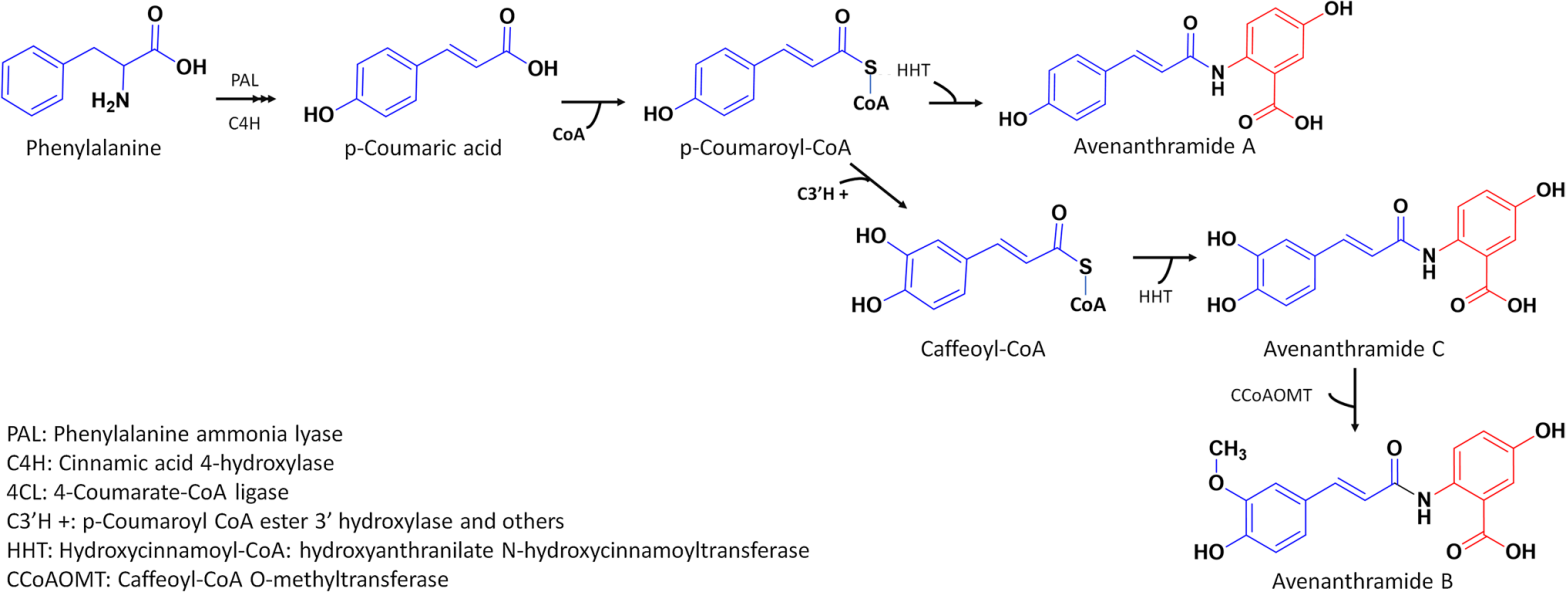
Suggested biosynthetic pathway of avenanthramides A, B and C in oat, which are made up of an anthranilic acid (AA, red) and a phenylalkenoic acid (PA, blue). The PA moiety originates from the early phenylpropanoid pathway via the stress-inducible PAL, while the anthranilic acid is synthesised by the shikimate pathway via chorismate. The conjugating enzyme HHT catalyses the N-acylation of hydroxyanthranilates with various activated hydroxycinnamoyl-CoAs as aroyl-moieties, resulting in a succession of avenanthramides. Thus, Avns are N-cinnamoylanthranilic acids, with *p*-coumaroyl-CoA producing Avn A and caffeoyl-CoA producing Avn C. Avn B is formed via methylation of Avn C (adapted from Pretorius and Dubery 2023)

Recently, novel insights of how plants respond to biological stresses have been uncovered by using a combination of transcriptomics and metabolomics analyses that pinpointed pivotal genes, proteins and metabolites involved in specific plant–pathogen interactions (Kim et al. 2021b; Lemcke et al. 2021; Li et al. 2022). Although a growing amount of research utilising a multi-omics approach for studying plant–pathogen interactions has been applied, such studies on oat plant defences, especially inoculated with *P. syringae*, are scarce. Here, a targeted transcriptomic and metabolomic analysis was performed on the two oat cultivars, (Dunnart, tolerant to *Ps-c*, and SWK001, susceptible to *Ps-c*) inoculated with the respective *P. syringae* pathovars (pathogenic or non-pathogenic). Among these were *Ps-c,* a pathogen on oat causing halo blight disease; *P. syringae* pv. *tabaci* (*Ps-t*), the causing agent of tobacco wildfire disease; *P. syringae* pv. *tomato* DC3000 (DC3000) which causes bacterial speck disease on tomato and Arabidopsis; and *P. syringae* pv. *tomato* DC3000 *hrcC*^*−*^ mutant (*hrcC*^*−*^) which is deficient in T3SS (Persson and Sletten 2001; Guo et al. 2009, 2017).

The extensively researched host resistance is frequently referred to as gene-for-gene resistance and is typically extremely specific to a particular genotype or cultivar of plant and against a certain pathogen. Nonhost resistance, on the other hand, is a broad-spectrum resistance displayed by the entire plant species against a given disease and is not pathogen specific. Nonhost resistance is multi-tiered with many barriers reliant on a specific host to prevent pathogen colonisation. These barriers range from preformed physical and chemical structures like cell walls, cuticles, and phytoanticipins, to induced defence responses like lignin accumulation, production of antimicrobials (phytoalexins), the hypersensitive response (HR), and induction of pathogenesis-related (PR) proteins (Gill et al. 2015; Panstruga and Moscou 2020; Xu et al. 2022). Among the pathovars employed in this study, *Ps-c* is known to cause a host response on these particular cultivars, while *Ps-t*, DC3000 and the *hrcC*^*−*^ mutant elicit a nonhost response. Among the nonhost responses, *Ps-t* can further be described as a type II nonhost response which phenotypically resembles a typical gene-for-gene interaction and leads to an HR. DC3000 and *hrcC*^*−*^ on the other hand can be categorised as a type I nonhost response. During a type I nonhost response, the nonhost pathogen is unable to overcome the plant-induced defences such as cell wall thickening, phytoalexin accumulation, and the accumulation of plant secondary metabolites (Mysore and Ryu 2004; Gill et al. 2015). Therefore, this study highlights metabolic differences in phytoalexin accumulation between host and nonhost responses and indirectly reflects the outcome of effector proteins on the plant defence response (i.e. pattern-triggered immunity (PTI) vs PTI and effector-triggered immunity (ETI)) by comparing DC3000 and the *hrcC*^*−*^ mutant (deficient in the T3SS). The aim of this study was thus to quantify metabolites involved in defence-related metabolic reprogramming such as the synthesis of Avns and associated precursors (previously identified as signatory biomarkers in an untargeted metabolomics study, Pretorius et al. 2022), and analyse the up- or down-regulation of selected genes involved in the synthesis of these phytoalexins.

## Materials and methods

### Oat plant cultivation

Oat seedlings from two cultivars (‘Dunnart’, tolerant to *Ps-c* and ‘SWK001’, susceptible to *Ps-c*), were grown under controlled conditions. Briefly, seeds were germinated and grown under greenhouse conditions (light/dark cycles of 12 h/12 h with a light intensity of ≈ 80 µmol m^−2^ s^−1^ and temperature set to 25 °C) in pasteurised (90 °C) soil (Culterra germination mixture, Muldersdrift, South Africa) and watered twice a week. Three biological replicates were grown for each condition (one biological replicate = one pot) and for every time point. At 3-week maturity (three-leaf stage), the plants were infected with the respective *P. syringae* pathovars.

### *Pseudomonas syringae* preparation and inoculation of oat seedlings

The pathogenic strain of *P. syringae* pv. *coronafaciens* was obtained from Dr. W. Kriel (Starke Ayres Seeds, Bredell, South Africa). *P. syringae* pv. *tabaci* w*a*s obtained from Prof. Y. Ichinose, Okayama University, Japan, and *P. syringae* pv. *tomato* DC3000 and its *hrcC*^−^ mutant from Dr. B. Kemmerling, University of Tuebingen, Germany. The identification of the respective pathovars (*Ps-c*, *Ps-t*, DC3000, *hrcC*^*−*^) was validated using 16S rRNA sequencing by Inqaba Biotechnical Industries (Pretoria, South Africa). The bacterial strains were grown overnight in nutrient broth at 28 °C on an orbital shaking incubator. The overnight cultures were diluted to an OD600 ≈ 0.3 using 0.1% Tween 20 and phosphate-buffered saline (PBS). The same dilution was used to make up the solution for the vehicle control (not containing the pathogen). The seedlings were infected at the three-leaf growth stage by spray inoculation (50 mL) with the respective inoculums (*Ps-c*, *Ps-t*, DC3000 and *hrcC*^*−*^). The vehicle control plants were sprayed with the solution (50 mL) free of the bacteria and the healthy/non-treated control groups were untreated (i.e. not sprayed with either solution). The inoculated seedlings were placed in a dark incubator for 1 h to provide 100% relative humidity. Following the initial incubation, the plants were sprayed with another 50 mL of either inoculum or control solution and further incubated for 6 h. The plants were then returned to the initial controlled conditions. Post-treatment harvesting was done at 2, 4 and 6 dpi and immediately frozen with liquid nitrogen to quench metabolic activity. Leaves were stored at −80 °C until metabolite extraction.

### Extraction of metabolites and sample preparation

Frozen leaf material was crushed with liquid nitrogen into powder form using a mortar and pestle. One gram of each sample was weighed out and suspended in 80% cold (4 °C) aqueous methanol at an m/v ratio of 1:10. The suspension was then homogenised using a probe sonicator (Bandelin Sonopuls, Berlin, Germany) and centrifuged at 5100×*g* for 20 min at 4 °C. The supernatants were kept and subsequently concentrated by evaporation to approximately 1 mL using a rotary evaporator set to 55 °C. The concentrated samples were transferred to 2 mL microcentrifuge tubes and dried to completion in a centrifugal evaporator under vacuum. The dried extracts were reconstituted in 500 μL of 50% aqueous methanol (LC-grade, Romil Pure Chemistry, Cambridge, UK) and filtered through nylon syringe filters (0.22 μm) into chromatography vials fitted with 500 μL inserts, capped, and kept at 4 °C until analysis.

### Sample analysis using UHPLC–QqQ-MS with multiple reaction monitoring

The authentic standards were purchased from BDH (Poole, UK) (tryptophan, tyrosine, phenylalanine, cinnamic acid, caffeic acid, ferulic acid, and sinapic acid), and Sigma-Aldrich (St. Louis, MO, USA) (avenanthramide A, avenanthramide B, and hordenine). Ultrahigh performance liquid chromatography (UHPLC) was used to separate 1 ppm (part per million) stock solutions of the pure standards and internal standard (D-fluorophenylalanine, Sigma-Aldrich) for optimisation, followed by triple quadrupole MS with electrospray ionisation (ESI) in MRM mode for quantification of the targeted compounds using calibration curves.

Standard solutions and sample extracts were analysed on a Shimadzu Nexera 30A UHPLC system linked to a Shimadzu 8030 mass spectrometer (Shimadzu, Kyoto, Japan) using a C18 reverse phase chromatography column (Restek AQ, 100 mm × 2.1 mm, 3 µm particle size) from Restek, (Bellefonte, PA, USA). The MS parameters were set as follows: 4.5 kV interface voltage, 2.75 Amp interface current, and 400 and 249 °C for the heat block and dissolvation temperature, respectively. Argon gas was employed as a nebulising gas, and the flow rate of the nitrogen gas was set to 15 L min^−1^. The MRM tool from Shimadzu’s ‘LabSolutions’ software was used for automated optimisation of the parameters for the precursor and representative product ion of each analyte, i.e. the MRM workflow was optimised with the second quadrupole (MS2) set to filter for unique quantifier and qualifier product ions only formed from the analyte (Table S1).

The concentrations of all standards were prepared as a pooled working solution where 100 ppm stocks were made and diluted in a 50% (v/v) UHPLC grade methanol/MilliQ Water solvent for the creation of calibration curves. From the stock solutions, serial dilutions were made to 4.5, 2.5, 1, 0.5, 0.25, 0.1, 0.05, and 0.025 ppm (1 ppm = 1 mg L^−1^). Each sample was injected in triplicate, and the standard solutions were generated in triplicate and analysed using the same column and UHPLC–QqQ-MS equipment as indicated previously. The acquired integrated peak area vs the standard concentration were plotted for the construction of the standard curves. Table S1 provides an overview of the standard curve equations and *R*^2^ values. Values in ppm were relayed to a dry weight of µg g^−1^ of plant material. Both the samples and the working solutions were analysed using a continuous flow rate of 0.4 mL min^−1^, and the injection volume was 1 µL. The standards and samples were injected in triplicate and separated using a binary gradient (solvent A: MilliQ Water with 0.1% (v/v) formic acid, solvent B: UHPLC grade methanol with 0.1% (v/v) formic acid). In the binary gradient, the concentration of solvent B was increased by 5% (v/v) increments to 25% (v/v) at 2–18 min and 95% (v/v) at 25–30 min before being reduced to 2% at 31 min.

**Fig. 2:**
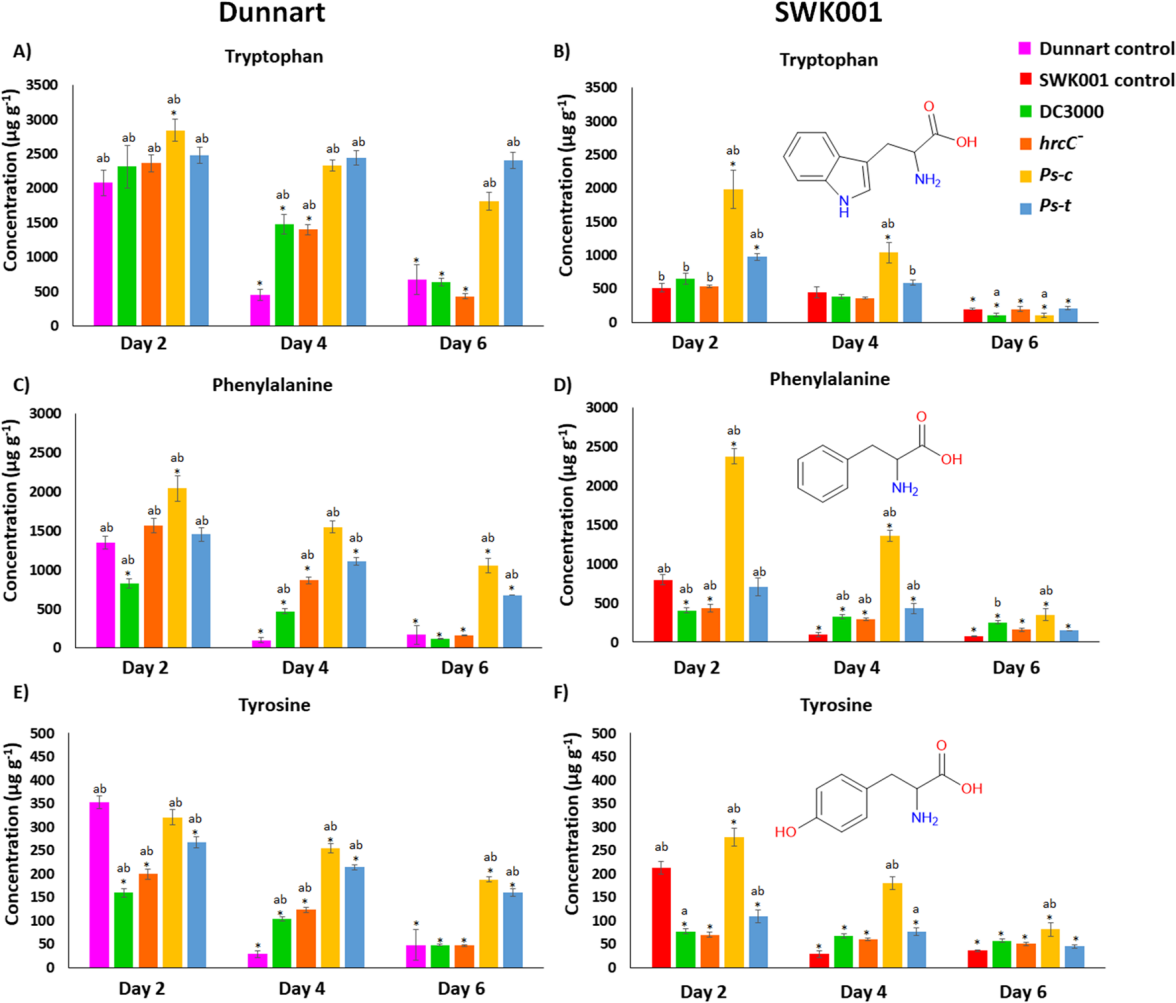
MRM quantification of aromatic amino acids in Dunnart and SWK001 oat seedlings under treatment with the respective *P. syringae* pathovars. The bar graphs represent averages of the concentrations in the control and treated groups calculated for Dunnart (**A**, **C** and **E**) and SWK001 (**B**, **D** and **F**) using three biological and three technical replicates (*n* = 9). Concentrations of tryptophan (**A** and **B**), phenylalanine (**C** and **D**), and tyrosine (**E** and **F**) are shown

**Fig. 3:**
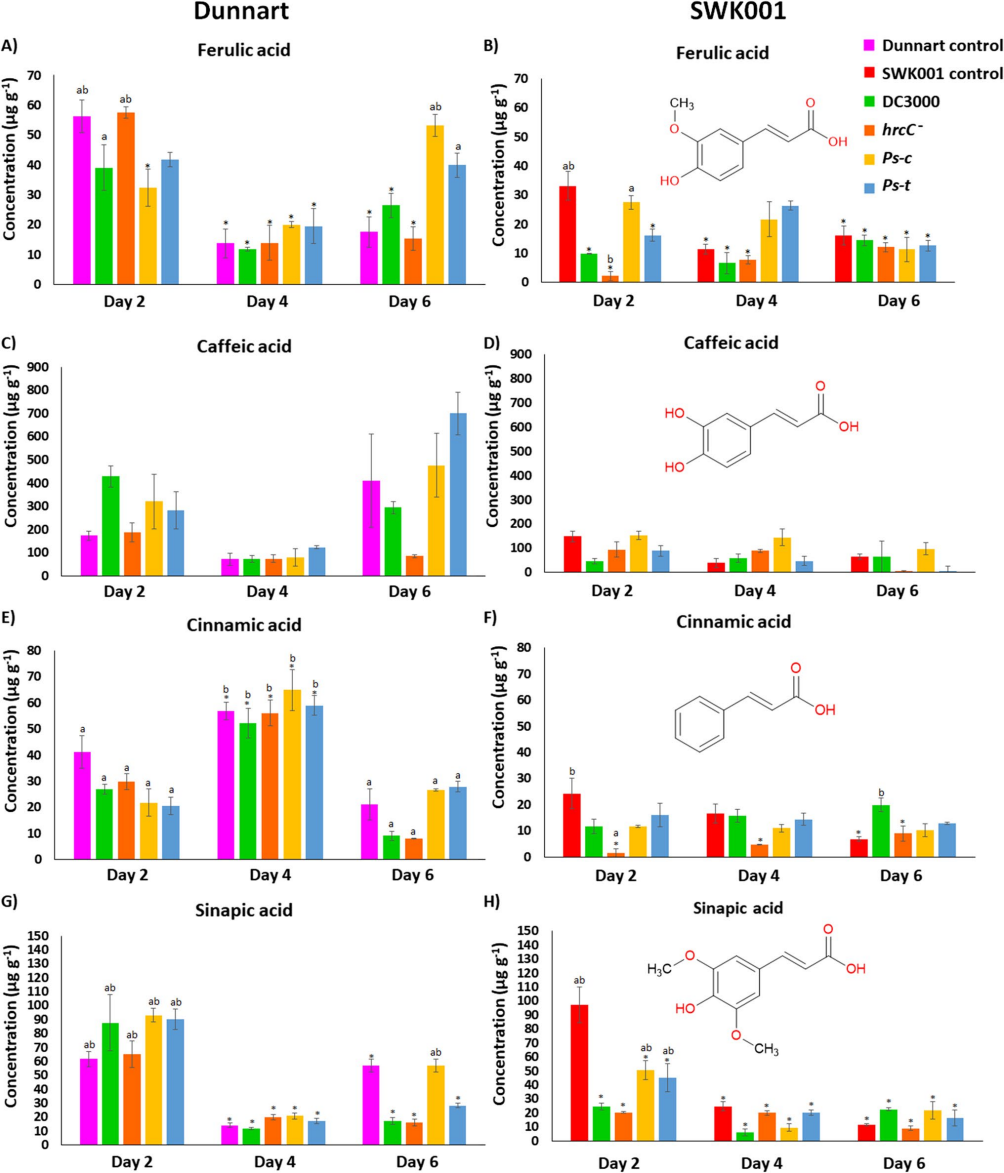
Quantification of HCAs present in Dunnart and SWK001 cultivars under treatment with the respective *P. syringae* pathovars. The bar graphs represent averages of the concentrations in the control and treated groups calculated for Dunnart (**A**, **C**, **E** and **G**) and SWK001 (**B**, **D**, **F** and **H**) using three biological and three technical replicates (*n* = 9). Concentrations of ferulic acid (**A** and **B**), caffeic acid (**C** and **D**), cinnamic acid (**E** and **F**), and sinapic acid (**G** and **H**) are shown

**Fig. 4:**
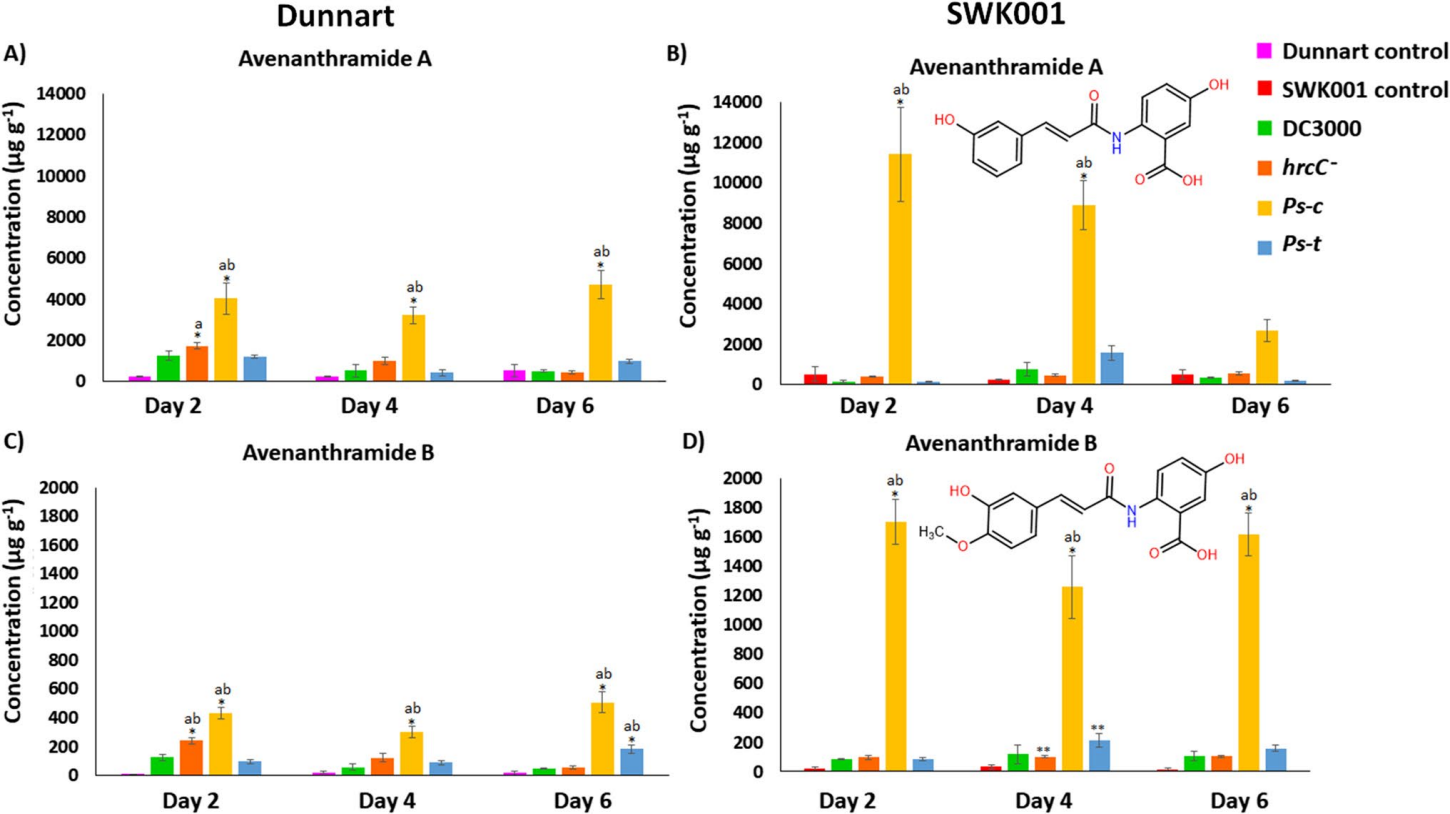
Quantification of Avns present in Dunnart and SWK001 under treatment with the respective *P. syringae* pathovars. The bar graphs represent averages of the concentrations calculated using three biological and three technical replicates (*n* = 9). **A** Concentration(s) of Avn A in Dunnart control and treated groups. **B** Concentration(s) of Avn A present in SWK001 control and treated groups. **C** Avn B concentrations in the Dunnart control and treated groups. **D** Avn B concentrations present in SWK001 control and treated groups

**Fig. 5:**
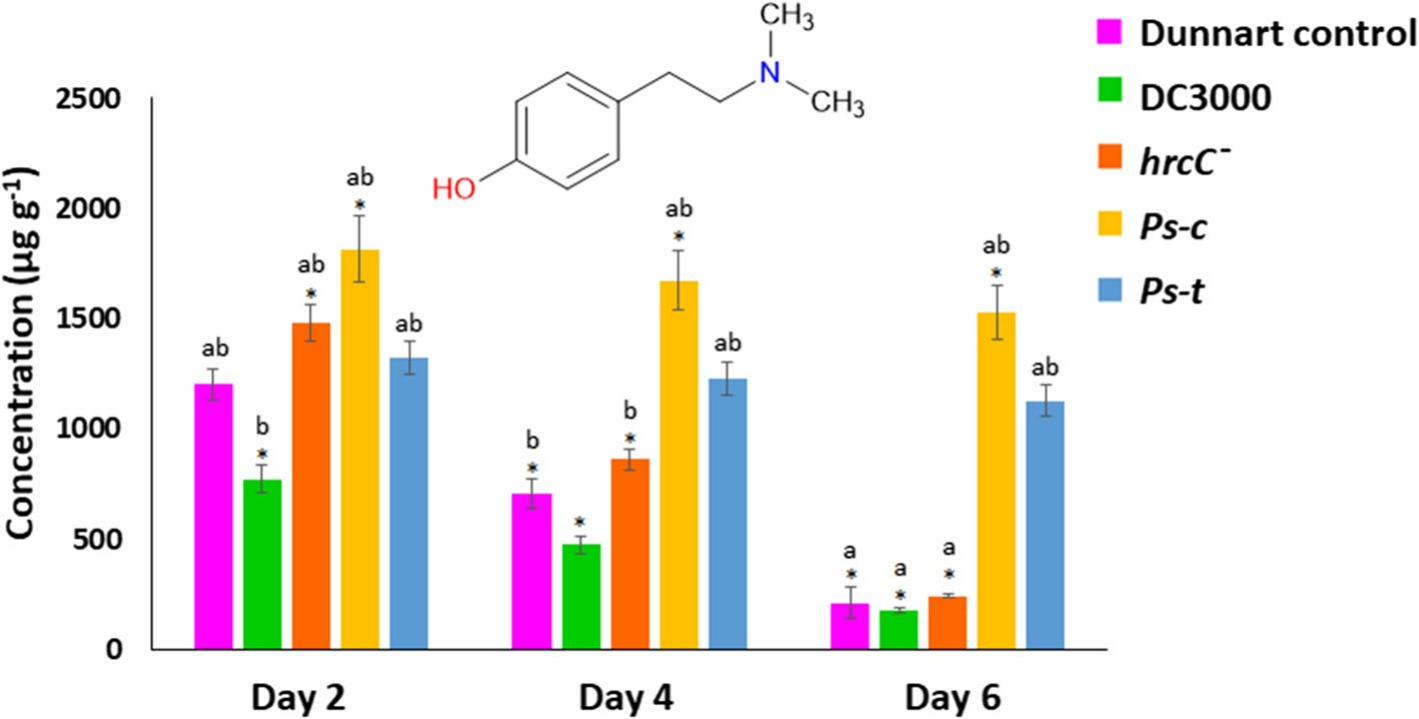
Quantification of hordenine present in the Dunnart cultivar under treatment with the respective *P. syringae* pathovars. The bar graphs represent averages of the concentrations calculated using three biological and three technical replicates (*n* = 9)

Univariate analysis of variance (ANOVA) in two-tailed complete randomised blocks was used to compare non-treated (control) and *P. syringae*-infected plants at various time points. ANOVA was followed by the Tukey post hoc test (IBM® SPSS® version 29.0.2.0). Differences between means were considered significant at *P* < 0.05, as indicated in graphs (Figs. 2, 3, 4, 5) with an asterisk (*) or the letters ‘a’ and ‘b’. Asterisks (*) indicate significant differences between the day 2 control and day 2, 4, or 6 treatment groups. Similarly, ‘a’ indicates significant differences between the day 4 control and the treatment groups. The letter ‘b’ denotes a statistically significant difference between the day 6 control and treatments. The error bars represent the standard error of the mean. Concentrations are expressed in μg g^−1^ dry weight (average ± standard deviation).

### RNA extraction and qRT-PCR analysis

Leaf samples from Dunnart and SWK001 (either treated with *Ps-c* or non-treated control) from 1 to 6 dpi were used to extract total RNA following the protocol of the Zymo Quick RNA Plant kit (Zymo Research, Irvine, CA). An earlier time point (1 dpi) was included in the expression analysis to detect early responses. Biological replicates of each sample were pooled and extracted, i.e. 80 mg of each biological replicate of each sample was pooled into one tube for RNA extraction. The quality and concentration of RNA samples were measured using a NanoDrop™ One Microvolume UV–Vis Spectrophotometer (Thermo Fisher Scientific, Waltham, MA, USA). First-strand cDNA was synthesised using LunaScript RT Super mix kit (New England Biolabs, Ipswich, MA, USA) according to manufacturer’s instructions in a total volume of 20 µL containing 1 µg total RNA. Quantitative RT-PCR was then performed in 96-well plates with Luna Universal qPCR Master Mix (New England Biolabs) using dye-based qPCR assay. Plates were divided by cultivar, all samples from each cultivar were run on the same plate, i.e. controls and treated samples of each cultivar. Two house-keeping genes (9-HK: ADP-ribosylation factor, ADPR, primers designed using Primer-BLAST, and 10-HK: glyceraldehyde-3-phosphate dehydrogenase, GAPDH, primer sequences taken from Tajti et al. 2021) were run with three targets for a total of five targets per plate. Each reaction contained 1 µL of cDNA template, 0.25 µM forward and reverse primers, and 1X Luna Universal qPCR Master mix (primers are listed in supplementary Table S1). Primer sequences (1–3 and 6) were taken from Kim et al. (2021a). Primer sets 4, 5, 7, and 9 were designed using Primer-BLAST available on NIH (https://www.ncbi.nlm.nih.gov/tools/primer-blast/). Primer sets 8 and 10 were taken from Bahraminejad (2007) and Tajti et al. (2021), respectively. The reactions were run on CFX96 Real-Time PCR System (Bio-Rad, Hercules, CA, USA) following a standard two-step PCR programme as suggested by Luna Universal qPCR Master Mix manual. Three technical replicates were run for each cDNA sample. Amplification of different input templates was evaluated based on quantification cycle (Cq) value. The reactions were subjected to an initial denaturation step at 95 °C for 60 s, followed by 45 cycles of denaturation at 95 °C for 15 s, and annealing at 60 °C for 30 s, with melting curve analysis performed from 60–95 °C in increments of 0.2 °C for 10 s. Transcript levels were calculated by normalisation against ADP-ribosylation factor (AB050957) as a reference control. Relative changes in gene expression levels were determined by the 2(−delta delta C(T)) or 2^–∆∆C^_T_ method (Livak and Schmittgen 2001).

## Results

### Targeted metabolite analysis using UHPLC–QqQ-MS

Aromatic amino acids act as precursors for a range of defence-related compounds including hydroxycinnamic acids (HCAs), flavonoids, and alkaloids. Here these amino acids were quantified in the susceptible and tolerant oat cultivars (SWK001 and Dunnart) inoculated with the respective *P. syringae* pathovars using an MRM method developed on a UHPLC–QqQ-MS system. Leaf extracts from the two cultivars were used to compare the non-treated control (control) to the individually treated groups (DC3000, *hrcC*^*−*^, *Ps-c* and *Ps-t*) across 2–6 days post-inoculation (dpi). In both treated and control groups the aromatic amino acids reached maximum concentrations at 2 dpi and decreased over time at 4 and 6 dpi. Decreases in the concentrations might be attributed to the fact that these amino acids act as precursor metabolites in support of enhanced synthesis of secondary metabolites. The highest concentrations were noted in the *Ps-c* and *Ps-t*-treated plants compared to the controls in both cultivars (Fig. 2). In most cases, changes in concentrations between the respective time points of the control and treated groups were noted to be significant following a one-way ANOVA that compared the mean values of quantified metabolites in control vs treated groups and the Tukey post hoc test. Tryptophan and phenylalanine concentrations were higher for both cultivars compared to tyrosine. In general, concentrations were often higher in the treated plants compared to the control, except at 2 dpi where the nonhost pathogens (DC3000 and *hrcC*^*−*^ mutant)-treated groups often had lower concentrations of the respective amino acids compared to the control.

HCAs can be broadly categorised as metabolites consisting of a cinnamic acid carbon skeleton and include coumaric, caffeic, ferulic, and sinapic acids. These phenolic compounds are some of the most abundant naturally occurring metabolites. As was already established, these metabolites play a crucial role as precursors in the production of Avns in oats (Taofiq et al. 2017; Lee et al. 2018; Pretorius et al. 2022). For this reason, these HCAs have been quantified here using the described MRM method. Overall, the concentrations of ferulic, caffeic, and sinapic acid in the Dunnart cultivar (Fig. 3A, C, E and G) appear to be higher in both control and treated groups at 2 and 6 dpi. Cinnamic acid on the other hand appears to reach maximum concentration at 4 dpi. Ferulic acid was significantly lower in the DC3000 (0.7-fold), *Ps-c-* (0.6-fold) and *Ps-t-* (0.7-fold) treated groups in the Dunnart cultivar at 2 dpi, whereas the *hrcC*^*−*^ mutant showed no significant difference compared to the day 2 control group. The only other significant increase can be seen for the *Ps-c* (3-fold) treatment at 6 dpi for this cultivar. SWK001 showed no particular trend in fluctuations of the HCAs (Fig. 3B, D, F and H). Ferulic acid concentrations can be seen as significantly decreasing in SWK001 at 2 dpi (DC3000, *hrcC*^*−*^and *Ps-t* by 0.3-, 0.1-, and 0.5-fold) compared to the control with no significant changes at 4 dpi. Cinnamic acid similarly showed a significant decrease in the SWK001 cultivar at 2 dpi for DC3000, *hrcC*^*−*^, and *Ps-c* (0.5-, 0.1-, and 0.5-fold) and at 4 dpi for *Ps-c* (0.7-fold). At 6 dpi, however, an increase was noted for the DC3000 (2.9-fold) treatment compared to the day 6 control. In the Dunnart cultivar, no significant changes were noted except for the down-regulation of DC3000 and *hrcC*^*−*^ (0.4- and 0.38-fold) at 6 dpi. Furthermore, sinapic acid showed no significant changes compared to the day 2 control in Dunnart at 2 dpi; however at 6 dpi, the concentrations were significantly down-regulated (0.3-, 0.3-, and 0.5-fold) for the different treatments (DC3000, *hrcC*^*−*^, and *Ps-t*). In the SWK001 cultivar significantly lower concentrations (0.3-, 0.2-, 0.5- and 0.46-fold) of sinapic acid were present in the treated groups (DC3000, *hrcC*^*−*^, *Ps-c* and *Ps-t*) at 2 dpi compared to the day 2 control and at 4 dpi (0.2, 0.8, 0.4 and 0.8).

As described in previous studies (Pretorius and Dubery 2023), the oat-specific Avns are produced in response to pathogen infection and function as phytoalexins that are induced to act as chemical defence antimicrobials and substrates for cell wall reinforcement. Furthermore, these phytoalexins were identified as discriminatory metabolites in the treated groups of both cultivars and were therefore quantified here to determine the content of these metabolites under treatment with the respective *P. syringae* pathovars. As clearly noted (Fig. 4), concentrations were most abundant in the *Ps-c*-treated groups (host response) compared to the other treatments in both cultivars. Dunnart presented 16-, 14- and 9-fold increases in Avn A at 2, 4, and 6 dpi, respectively, for the *Ps-c* treatment compared to the control (Fig. 4A). All treatments showed significant increases in Avn A content at 2 dpi with 5-, 7-, 16- and 5-fold increases for DC3000, *hrcC*^*−*^, *Ps-c,* and *Ps-t,* respectively; however at 4 dpi, only *Ps-c* and *hrcC*^*−*^ (4-fold) showed significant increases in Avn A content. The SWK001 cultivar similarly showed significant increases in Avn A content at all time points for the *Ps-c* treatment with 22-, 36-, and 5-fold increases compared to the control. The only other significant increase was noted for *hrcC*^*−*^ (2-fold) and *Ps-t* (6-fold) at 4 dpi (Fig. 4B). Avn B again showed significant up-regulation for all treatments in the Dunnart cultivar at 2 dpi with 50-, 97-, 175- and 38-fold increases compared to the control. At 4 dpi, significant increases were noted for *hrcC*^*−*^, *Ps-c*, and *Ps-t* with fold changes of 6-, 14-, and 4-fold respectively; however at 6 dpi significant changes were observed in *Ps-c* and *Ps-t* only (33- and 12-fold, respectively) (Fig. 4C). Similarly, SWK001 showed the greatest fold changes after treatment with *Ps-c* for 2, 4, and 6 dpi with 89-, 39- and 115-fold increases, respectively (Fig. 4D). Due to the significant increase in both Avn A and B content (Dunnart and SWK001) after treatment with *Ps-c*, qRT-PCR was used to measure the expression levels of genes encoding enzymes (PAL, 4CL, CCoAOMT, and HHT) involved in the Avn biosynthesis pathway for both these cultivars (Sect. "*Pseudomonas syringae* preparation and inoculation of oat seedlings").

Hordenine has numerous bioactivities in plants, particularly as an antimicrobial phenethylamine alkaloid. This particular metabolite was initially discovered in barley and has since been detected in a variety of grass crops. It has also been demonstrated to have a role in plant defence responses by activating jasmonate-dependent pathways (Ishiai et al. 2016). Hordenine was also identified as a discriminant feature among the treated groups in the Dunnart cultivar in preliminary untargeted analyses and was not detected in the SWK001 cultivar. Upon treatment with *hrcC*^*−*^ and *Ps-c*, an increase in hordenine content was noted (Fig. 5). This increase was significant at 2 dpi with fold changes of 1.2- and 1.5-fold for the treatments, respectively. DC3000 on the other hand showed a 0.6-fold decrease at this time point and *Ps-t* was unchanged. At 4 dpi, increases of 1.2-, 2.4- and 1.7-fold were noted for *hrcC*^*−*^, *Ps-c*, and *Ps-t*. Furthermore, at 6 dpi, significant increases were only noted for *Ps-c* and *Ps-t* with 7.2- and 5.3-fold increases after treatment with these pathovars compared to the control.

### qRT-PCR expression analysis of avenanthramide biosynthetic genes in *Ps-c* inoculated oat seedlings

**Fig. 6:**
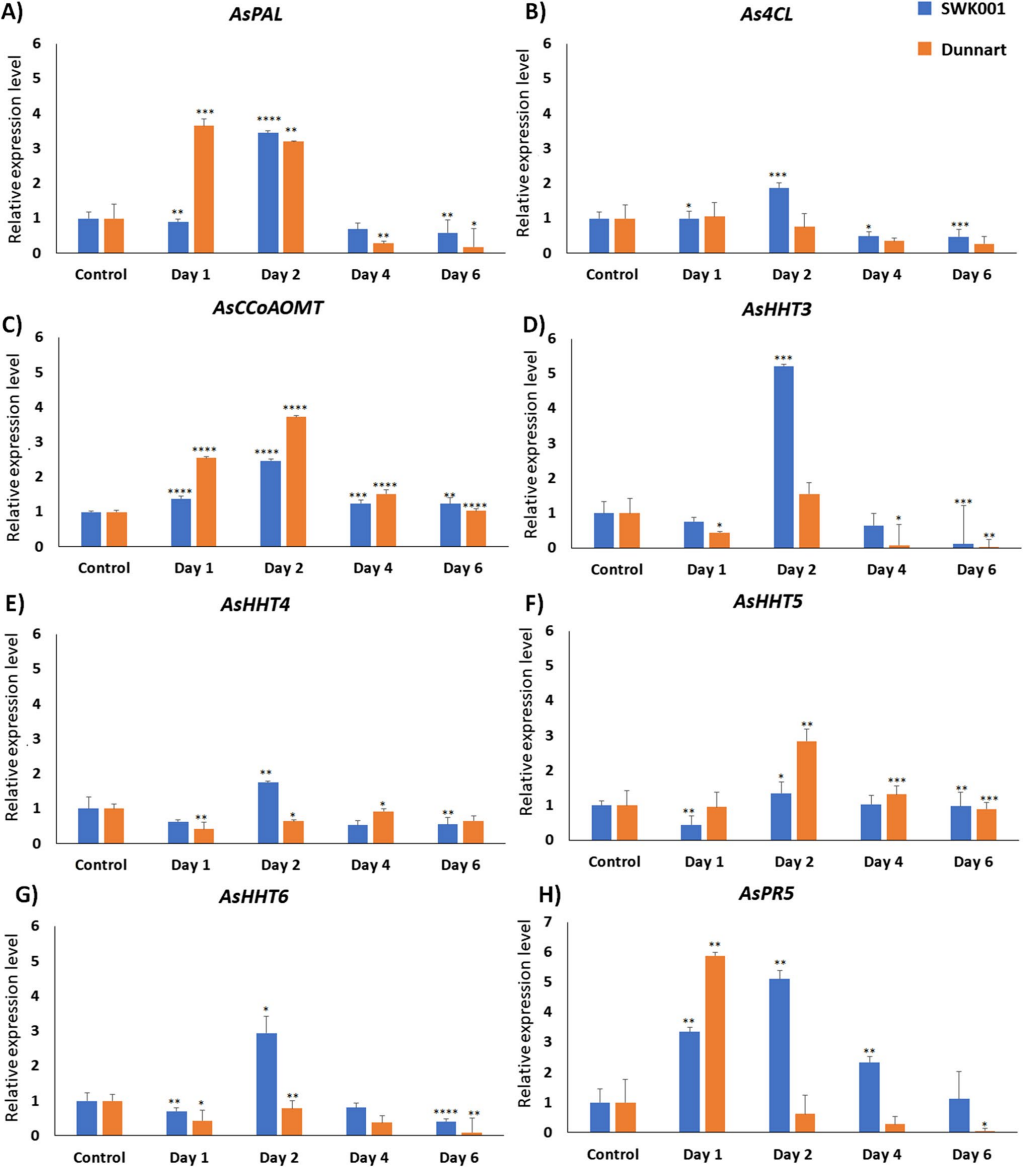
Relative expression levels of genes associated with avenanthramide biosynthesis in the two oat cultivars treated with *P. syringae* pv. *coronafaciens*. Transcript levels of SWK (susceptible phenotype, blue) and Dunnart (tolerant phenotype, orange) include: **A** *AsPAL*, **B** *As4CL*, **C** *AsCCoAoMT*, **D** *AsHHT3*, **E** *AsHH4*, **F** *AsHHT5*, and **G** *AsHHT6,* analysed by qRT-PCR at 1, 2, 4, and 6 dpi. *AsPR5* (**H**) is not related to the biosynthesis, but is included as a defence-related marker. Relative expression levels were normalised against AsADP-ribosylation factor (AB050957). Error bars represent standard deviation. Student’s *t* test: *P* values < 0.05 (*), 0.01 (**), 0.001 (***), and 0.0001 (****)

Quantitative reverse transcription PCR (qRT-PCR) was performed to determine the expression levels of the genes encoding the PAL, 4CL, CCoAOMT, and HHT1-6 enzymes that are involved in the synthesis of Avns, with the ADP-ribosylation factor (AB050957) gene serving as an internal reference. These expression levels were determined from leaves of the two oat cultivars treated with *Ps-c* and compared to non-treated control plants (Fig. 6). The highest expression of *AsPAL, As4CL, AsCCoAoMT, AsHHT3, -4, -5, -6* and *AsPR5* for the SWK001 cultivar was observed at 2 dpi. This was positively correlated with the high Avn production observed in the SWK001 cultivar at 2 dpi (Fig. 4B and D). The highest expression levels in the Dunnart cultivar were observed at 1 dpi for *AsPAL* and *AsPR5. AsCCoAoMT* on the other hand, showed a higher level of expression at 2 dpi. The results further showed that *AsPAL* was up-regulated for SWK001 at 2 dpi (3-fold) and down-regulated at days 1, 4, and 6 (0.9-, 0.7- and 0.6-fold). Dunnart on the other hand showed an up-regulation at day 1 and 2 of 3.7- and 3.2-fold, respectively; however, at days 4 and 6, expression of this gene was down-regulated (0.3- and 0.2-fold). Moreover, *As4CL* expression was up-regulated 2-fold at 2 dpi and down-regulated at days 4 and 6 (0.5-fold) for SWK001, and for Dunnart a decrease can be seen from days 2 to 6 (0.8-, 0.4- and 0.3-fold) following treatment. *AsCCoAoMT* expression increased for SWK001 from 1 to 6 dpi (1.4-, 2.5-, 1.3- and 1.2-fold) and in Dunnart from 1–4 dpi (2.5-, 3.7-, 1.5-fold), with no change noted at 6 dpi relative to the control. Furthermore, *AsHHT3* expression was increased by 5-fold in SWK001 (2 dpi) but reduced to 0.8-, 0.7-, and 0.1-fold in days 1, 4, and 6 post-inoculation compared to the control. Similarly, Dunnart only exhibited an increase at 2 dpi (1.6-fold) and showed subsequent down-regulation for days 1, 4, and 6 (0.4, 0.1-, and 0.03-fold). *AsHHT4* expression was up-regulated 2-fold in SWK001 (2 dpi), but down-regulated 0.6-, 0.5, and 0.5-fold in days 1, 4, and 6. Dunnart was down-regulated at all time points (0.4-, 0.6-, 0.9- and 0.7-fold) for *AsHHT* after treatment with *Ps-c*. However, *AsHHT5* expression was increased following treatment in Dunnart at 2 and 4 dpi (2.8- and 1.3-fold); furthermore, SWK001 also showed up-regulation (1.4-fold) at day 2. *AsHHT6* only showed up-regulation (2.9-fold) for SWK001 at 2 dpi compared to the control. Among all the *AsHHTs* examined, the greatest increase in expression in response to *Ps-c* treatment was observed in *AsHHT3* in SWK001 and *AsHHT5* in Dunnart; therefore, this could be indicative of *AsHHT3* and *AsHHT5* playing an important role in Avn biosynthesis in oats in response to *Ps-c* treatment. The expression level of *AsPR5* was also analysed here since this particular gene (*PR-5*, also known as thaumatin-like protein) is not involved in Avn synthesis, but has been known to play an important role in response to pathogen infection. Comparison between the two cultivars showed the highest expression of *AsPR5* was observed in Dunnart at 1 dpi (5.8-fold) and in SWK001 (5-fold) at 2 dpi.

## Discussion

Major advances in the study of plant secondary metabolism and specialised metabolites have shifted the focus from ‘what’ to ‘where, how, and why’ (Dixon and Dickinson 2024). The redirection of primary metabolism to defence metabolism (Hamany Djande et al. 2023a) is the consequence of changes in regulatory pathways (originating from intricate signal perception and transduction events), and from increasing demands for energy and biosynthetic capacity required for defence. Research based on technological advances is increasingly applying integrated omics approaches to perform molecular investigations on plant–pathogen interactions (Chen et al. 2018). The results from these analyses are then ultimately applied and incorporated into breeding programmes which allows for the selection of markers and genes related to plant resistance against pathogens (Yang et al. 2021).

In this study, a targeted metabolomics approach was applied to quantify important defensive metabolites involved in the *A. sativa*–*P. syringae* interactions. The Avns are central hydroxycinnamic acid amides (HCAAs) in the small molecule defence arsenal of oat, are primarily produced in vegetative tissue and the grain, and strongly influenced by environmental conditions (Wise 2018). Among the pathovars, differences in the magnitude of Avn production were noted between the host (*Ps-c*) and nonhost (*Ps-t*, DC3000 and *hrcC*^*−*^) responses. The most significant increase in Avn production was noted for *Ps-c* treatment in both cultivars across the time points compared to the nonhost responses. For the nonhost responses, similar increases and trends were noted for *Ps-t* and DC3000; *hrcC*^*−*^ on the other hand produced the second greatest magnitude of Avn in Dunnart. In the SWK001 cultivar, *Ps-c* was followed by *Ps-t* for the greatest production of these phytoalexins. Nevertheless, all pathovars did induce the production of the Avns, indicating shared underlying mechanisms among host and nonhost responses. Despite the fact that host and nonhost responses overlap, the latter is regarded to be a more complex mechanism of plant resistance and may vary depending on the nature of the pathogen and the host. This type of resistance is often thought to be a more durable form of defence as it provides broad-spectrum resistance to a range of microbes compared to the typical gene-for-gene resistance (Li et al. 2012). Among the nonhost responses, the DC3000 (PTI and ETI) and its *hrcC*^*−*^ mutant (deficient T3SS) counterpart (PTI) showed different responses, with *hrcC*^*−*^ often producing a greater amount of Avns compared to the DC3000. This suggests that the initial PAMP recognition is sufficient to induce the production of these metabolites. Since PTI is activated in the *hrc-*treated plants and not ETI due to the lack of the T3SS, the higher magnitude of these phytoalexins could be attributed to effectors secreted by DC3000 (via T3SS) able to suppress the PTI defence response and hence dampening the amount of the phytoalexins produced. Some effectors such as the AvrE type have been associated with causing perturbations to the phenylpropanoid pathways that can disrupt downstream production of secondary metabolites, suppress host defences, and suppress the HR to promote bacterial growth. One such study was done on maize metabolism using AvrE effectors from *Pantoea stewartii* (Asselin et al. 2015). However, the total defence response is multifaceted and cannot be explained only by the magnitude of the induction of the defensive compounds.

**Fig. 7:**
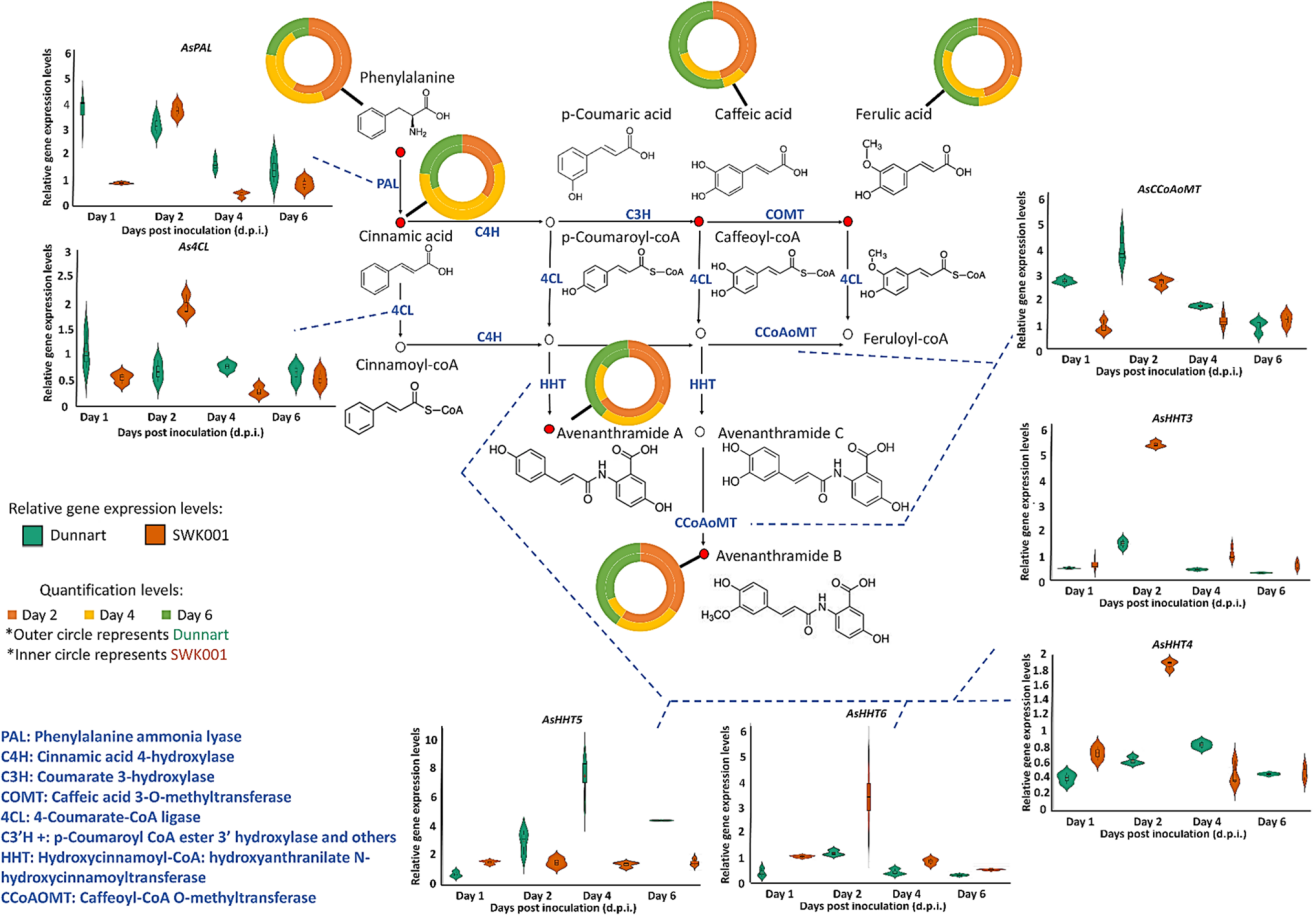
Avenanthramide biosynthesis pathway in leaf tissue of oat seedlings treated with *Ps-c*. Shown are the gene expression levels of genes encoding PAL, 4CL, CCoAOMT, and HHT3–6 enzymes in the form of violin plots, and the respective quantities of the metabolites involved in Avn biosynthesis for both Dunnart and SWK001 treated with *Ps-c*

Since it was noted that *Ps-c* treatment yielded the greatest concentration of Avns in both cultivars, subsequent transcript profiling was then applied to study the relative expression levels of genes encoding important enzymes involved in Avn synthesis in the tolerant and susceptible oat cultivars after treatment with *Ps-c*. Aromatic amino acids like tryptophan, phenylalanine, and tyrosine function as key components of primary metabolism in plants but also play a pivotal role as precursors for a wide range of plant secondary metabolites (Tzin and Galili 2010). In this regard, the phenylpropanoid pathway is crucial as it supplies the precursors required for the synthesis of a variety of secondary metabolites, including flavonoids and HCAs (Fraser and Chapple 2011). In plants, HCAs act as precursors of both inducible and constitutive defence metabolites. HCAs such as ferulic acid, caffeic acid, *p*-coumaric acid and sinapic acid were included here due to their antibacterial activity and their involvement in the synthesis of Avns (Liu et al. 2022). Since the greatest content of Avn A and B was observed after *Ps-c* treatment in both cultivars, these changes are thus further discussed. In the summary figure below (Fig. 7), the biosynthesis pathway of Avns in oat is demonstrated with relative gene expression levels and quantities of the metabolites involved in this pathway in both Dunnart and SWK001 treated with *Ps-c*. Surprisingly, while the trends in aromatic amino acids and HCAs seem to indicate stronger activation and flux through these pathways in Dunnart as compared to SWK001, the Avn results did not show a correlation between the accumulation of Avn and the tolerance *vs* susceptibility of the two cultivars. Here it is important to note that phytoalexins are only one component of the complex mechanisms for disease resistance in plants, and it is known that susceptible cultivars frequently maintain the ability to synthesise antimicrobial agents to the same extent as resistant lines. Accordingly, it has been proposed that it is the timing and initial magnitude of the response that play a determining role (Kuć and Rush 1985). Moreover, if the infection is not contained, the triggering signals for phytoalexin synthesis associated with the presence of the pathogen would remain/increase and lead to high(er) phytoalexin levels.

As described, the first step in the Avn biosynthesis pathway is initiated with the synthesis of trans-cinnamic acid from phenylalanine (Fig. 1). The phenylalanine content showed the highest levels for both cultivars at 2 dpi and subsequently decreased at 4 and 6 dpi, moreover the phenylalanine content was higher at every time point when treated with *Ps-c* compared to the untreated control. The observed decrease in phenylalanine across the time points in the treated groups could be explained by the conversion to cinnamic acid catalysed by PAL (an inducible, stress-responsive enzyme). The relative expression of *AsPAL* was greater at 1 dpi for the Dunnart cultivar compared to the SWK001 cultivar where increased content was noted at 2 dpi. Furthermore, in Dunnart the relative expression level remains high at 1- and 2 dpi and only starts decreasing considerably at 4 dpi, whereas in SWK001 a rapid increase was noted at 2 dpi followed by a rapid decrease at 4 dpi. This could possibly mean that the conversion to cinnamic acid is initiated earlier in Dunnart and that the conversion continues over a longer period compared to SWK001. The decrease in transcript levels of PAL can be explained as negative feedback inhibition at pre- and post-transcriptional levels to return the cellular state to a new homeostasis. Following this initial reaction, the *p*-coumaric acid is then converted into its activated CoA thioester analogue, by 4-coumarate-CoA ligase (4CL). *As4CL* was slightly up-regulated for SWK001 at 2 dpi and subsequently decreased over time, whereas in Dunnart a decrease was noted subsequent to day 1. The resulting *p*-coumaroyl-CoA is then conjugated to 5-hydroxyanthranilic acid, catalysed by HHT, to generate Avn A. Here, it was found that the expression of the *AsHHT3–6* genes was induced in both cultivars under *Ps-c* treatment; however, SWK001 was observed to have a higher expression of HHT3, 4, and 6, whereas Dunnart showed a higher expression of HHT5. In a study by Kim et al. (2021a), it was suggested that *AsHHT5* has a more crucial role in Avn synthesis compared to the other *AsHHT* genes. The quantitative data results show that Avn A is up-regulated compared to the control at all time points for both cultivars. Across the respective time points it is observed that Avn A content decreases from day 2 to day 4 followed by a slight increase again at day 6 for Dunnart and an overall decrease in SWK001. This can possibly be attributed to degradation, oxidation, conjugation, dimerisation or interconversion of Avn A, e.g. being used in plant defence by incorporation into the cell wall to provide protection against breakdown, which frequently happens as a result of enzymes released by pathogens (Ishihara et al. 2014; Pretorius et al. 2022).

Furthermore, Li et al. (2019) demonstrated that *AsHHTs* are involved in the synthesis the two major Avns (A and C) but not Avn B. The latter is believed to be synthesised by the CCoAoMT enzyme via O-methylation of Avn C. In this study, the highest content of Avn B was detected at day 2 post-inoculation in SWK001 and at day 6 in Dunnart. In both cultivars a similar trend is observed with higher concentrations at 2 dpi which then decreases at 4 dpi and again increases at 6 dpi. *AsCCoAoMT* expression was highest in Dunnart at 1- and 2 dpi, similarly SWK001 also showed greater expression at these time points with the expression decreasing towards day 6. Although Dunnart showed a greater expression of *AsCCoAoMT* at these time points, SWK001 exhibited higher levels of Avn B (Fig. 6C). Considering the fact that Avn B can be dimerised which could result in stronger antimicrobial activity than that of the original monomer and be incorporated into the cell-wall for strengthening, it could explain the decrease noted across the time points of the respective cultivars. Previous studies have shown that the Avn content of leaves and roots increases in response to pathogen infection and elicitor treatment (Wise 2011). Furthermore, Li et al. (2019) reported on the Avn content in oat grain and found Avn B to be present in higher concentrations compared to A and C. However, when it comes to treated leaves, Avn A was the most abundant of the Avns (Wise 2011). Ultimately, although Avn content is induced by pathogen treatment, various reports have concluded that the concentration and composition of these compounds are greatly influenced by factors such as genotype, environment, and growth conditions, and can thus vary among cultivars. It is also important to note that the increase in phytoalexin production may form part of a synchronised defence response, in which any one component may be insufficient to account for restriction of the potential pathogen. Furthermore, it is difficult to distinguish between phytoalexins produced for defence and those that result due to the pathogen-actuated stress metabolism since the metabolites are produced in both susceptible and resistant plants (Purkayastha 2017).

As mentioned, the expression level of *AsPR5* was also included in the RT-qPCR study, since the PR proteins are deployed by the host plant and are known to be induced by pathogen attack. The expression of these PR proteins is modulated by plant hormone networks like salicylic acid, jasmonic acid or ethylene. PR proteins have been extensively studied in cereals due to their well-established antifungal/antimicrobial properties. However, in oat there are limited reports on the expression of PR proteins in response to microbial inoculation. The absence of PR5 at constitutive levels in healthy plants and the fact that they are activated in response to injury or pathogen attack, make them ideal candidates for examining the activation of plant immunity. In this study, the highest expression of *AsPR5* was noted in the Dunnart cultivar at 1 dpi, SWK001 on the other hand showed an increasing level of expression from 1–2 dpi and subsequent decreases from 4- to 6 dpi. In a study by El-Kereamy et al. (2011) two *Prunus domestica* varieties, either resistant or susceptible to *Monilinia fructicola*, were studied in terms of their PR5 expression. It was determined that the resistant variety exhibited much higher levels of PR5 than the susceptible one. However, a significant increase in the transcript levels in the susceptible variety was still observed after infection. This correlates to the observed response for the Dunnart cultivar (tolerant to *Ps-c*) showing higher expression levels of PR5 compared to SWK001 (susceptible to *Ps-c*), but SWK001 still showing an increase in PR5 signifying the initiation of defence responses.

**Fig. 8:**
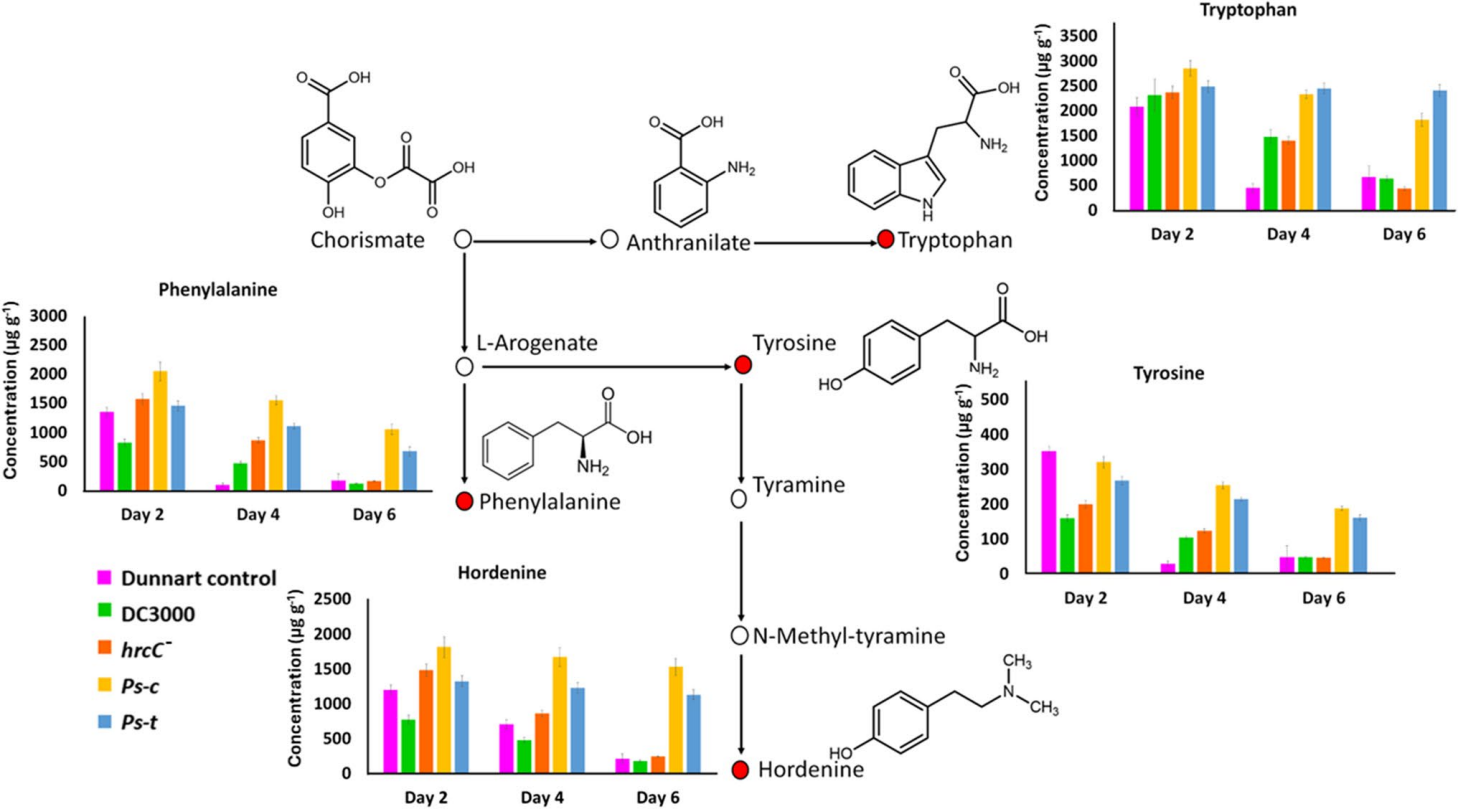
Aromatic amino acids pathway showing the origin of the synthesis of hordenine in the Dunnart cultivar under treatment with the respective *P. syringae* pathovars. Shown are the bar charts representing the quantified levels of tryptophan, phenylalanine, and tyrosine across 2–6 dpi after treatment with *Ps-t*, *Ps-c*, *hrcC*^*−*^, DC3000 and the untreated controls

Although not a member of the HCAAs class, hordenine plays an important role in plant defence as an allelochemical and antibacterial agent (Ishiai et al. 2016; Hamany Djande et al. 2023b). Hordenine is synthesised from tyrosine by the decarboxylation to tyramine followed by subsequent methylation to produce N-methyltyramine and ultimately hordenine, as illustrated below (Fig. 8). Hordenine content was greatest in Dunnart treated with *Ps-c* followed by treatment with *Ps-t*. Since hordenine was only successfully quantified in the Dunnart cultivar and not in SWK001, it could be suggested that this alkaloid significantly contributes to the tolerance of this cultivar to *Ps-c*. Additionally, in a study by Ishiai et al. (2016) it was noted that hordenine can be applied as an elicitor in plants to active jasmonate defence signalling pathways and can lead to the up-regulation of PR protein encoding genes.

## Conclusions

This study aimed at evaluating and quantifying metabolites involved in the synthesis of Avns as well as analysing the relative gene expression levels of genes involved in the synthesis of important enzymes necessary for the synthesis of these compounds. Furthermore, the relationship between the up- or down-regulation of the expressed genes and the respective quantities of the metabolites in the different cultivars were evaluated through transcript profiling using qPCR and MRM mass spectrometry. The results indicate that both the tolerant and susceptible oat cultivars treated with *P. syringae* pathovars induced the production of Avns in all treatments with the greatest abundance produced in *Ps-c*-treated plants. Furthermore, the correlation between Avn production and activation of Avn biosynthesis-related genes (*AsPAL*, *As4CL*, *AsCCoAoMT* and *AsHHT3–6*) in the seedlings treated with *Ps-c,* revealed a positive correlation between the up-regulation of *HHT* genes and a greater production of Avn A. The different responses to the *Ps-c* treatments and the subsequent differences in the synthesis of Avn A and B might also be attributed to genotypic differences between the two cultivars. Additionally, it can be deduced that overall tolerance or susceptibility of the respective cultivars to *Ps-c* is influenced by an array of defence metabolites and mechanisms and that the quantities of Avns present cannot predict higher tolerance or susceptibility outcomes on its own. However, the presence of these metabolites signifies induced defence responses upon treatment with the respective *P. syringae* pathovars and again reiterates the importance of these metabolites in oat plant defence and their function as antimicrobials. Future research is needed to cast a wider net in terms of the defensive metabolites and genes important in oat plant defence and resistance mechanisms especially to microbial pathogens since studies in this regard are scarce. These types of integrated studies can be particularly useful to describe the metabolic changes underlying these interactions. However, due to a range of limitations, such as the availability of authentic standards, the size and the number of genes known in the hexaploid oat genome, these types of studies are restricted. As a result, it is important to identify new metabolites and genes involved in oat defence responses to provide a more holistic understanding of the mechanisms involved in resistance of this important food crop to biotic stresses.

### Supplementary Information

Below is the link to the electronic supplementary material.

Supplementary file1 (DOCX 22 KB)

## Data Availability

The data reported in this study are contained within the manuscript and there is no associated data available.

## Abbreviations

Avn: Avenanthramide
*Ps-c*: *Pseudomonas syringae* pv. *coronafaciens*
*Ps-t*: *Pseudomonas syringae* pv. *tabaci*
DC3000: *Pseudomonas syringae* pv. *tomato* DC3000
*hrcC*^*−*^: *Pseudomonas syringae* pv. *tomato* DC3000 *hrcC*^*−*^ mutant
