# Supplementary material for: Integration of targeted metabolome and transcript profiling of Pseudomonas syringae-triggered changes in defence-related phytochemicals in oat plants
Source: Planta. 2024 May 24;260(1):8. doi: 10.1007/s00425-024-04435-w (PMC11126498; doi:10.1007/s00425-024-04435-w)
Supplement: Supplementary file 1 — Supplementary file1 (DOCX 22 KB) [file 425_2024_4435_MOESM1_ESM.docx]

**Integration of targeted metabolome and transcriptome profiling to quantify changes in molecular mechanisms underlying *Pseudomonas syringae*-based induced phytochemicals in oat crops**

Chanel J. Pretorius ^1^ and Ian A. Dubery ^,1^*

^1^ Research Centre for Plant Metabolomics, Department of Biochemistry, University of Johannesburg, P.O. Box 524, Auckland Park, Johannesburg 2006, South Africa; chanelpretorius5@outlook.com (C.J.P.).

* Correspondence: idubery@uj.ac.za; Tel.: +27-11-559240

**Supplementary file (Tables S1 and S2)**

**Table S1** Developed optimum parameters for Multiple Reaction Monitoring (MRM) analysis in ESI positive mode of the respective standards and their corresponding standard curve equations for quantification of the targeted oat compounds.

| **#** | **Standards** | **Precursor ion (*m/z*)** | **Product ions (*m/z*)** | **Rt (min)** | **CE (eV)** | **R^2^** | **Equation** |
| --- | --- | --- | --- | --- | --- | --- | --- |
| 1 | Avenanthramide A | 300.2877 | **283.1*>**  267.9>  43.1 | 17.081 | -5.0 | 0.996 | y=190363x+55209 |
| 2 | Avenanthramide B | 330.3078 | **281.1*>**  46.0>  43.2 | 17.493 | -6.0 | 0.996 | y=321208x+7455.4 |
| 3 | Ferulic acid | 195.1878 | 89.1>  **84.9*>**  45.0 | 12.418 | -8.0 | 0.998 | y=4×10^6^x+79059 |
| 4 | Caffeic acid | 181.1678 | **148.9*>**  116.9>  84.9 | 9.745 | -8.0 | 0.984 | y=236396x+23263 |
| 5 | Cinnamic acid | 149.1678 | **117.0*>**  84.9>  41.0 | 14.586 | -11.0 | 0.988 | y=824294x+10712 |
| 6 | Sinapic acid | 225.2178 | **207.0*>**  192.9>  174.8 | 12.885 | -6.0 | 0.999 | y=6×10^6^x+41962 |
| 7 | Hordenine | 166.2378 | **120.9*>**  103.0>  77.0 | 4.872 | -10.0 | 0.996 | y=3×10^7^x+351330 |
| 8 | Tryptophan | 205.2000 | **188.1*>**  146.2>  118.2 | 8.129 | -10.0 | 0.995 | y=4×10^7^x+393939 |
| 9 | Tyrosine | 182.0000 | 165.2>  **136.1*>**  91.2 | 3.772 | -13.0 | 0.993 | y=4×10^7^x+432965 |
| 10 | Phenylalanine | 166.0000 | **120.10*>**  103.20>  77.2 | 4.699 | -14.0 | 0.996 | y=1×10^8^x+2×10^6^ |
| 11 | D-Fluorophenylalanine  (Internal standard) | 184.0000 | **138.2*>**  118.2  91.2 | 5.304 | -14.0 | 0.997 | y= 9×10^7^x+283525 |

>: Transition, CE: Collision Energy, R: Regression coefficient.

*Using the authentic standards, the optimal ionisation polarity of the targets, the optimal MRM parameters, and optimization of MRM transitions were determined using the vendor-specific software, LabSolutions, (Shimadzu, Kyoto, Japan). The analyses were carried out using a triple quadrupole mass spectrometer with an ESI source that operated in both positive and negative ionisation modes. Shown is the ESI positive data since the compounds ionised optimally in this mode. The LC-MS/MS data obtained were analysed using the LabSolutions software. The MRM mode was employed to quantify the analytes using three transitions per compound. The product ions that were used for confirmation are indicated and the quantifier ions expressed in bold. Each calibration curve for the standards had a working concentration range of 0.025 ppm to 4.5 ppm, within which sample concentrations were collected. The R^2^ (regression) which is a measure of the standard curve's linearity based on the correlation between the independent (x-axis) and dependent (y-axis) variables are also indicated. The R^2^ values ranged from 0.984 to 0.999, indicating good linearity.

**Table S2** Primer pairs used for qRT-PCR analysis of induced expression of genes related to avenanthramide biosynthesis in oat, *A. sativa*.

| **#** | **Protein/Enzyme** | **Gene** | **Gene information** | **Uniprot ID** | **Primer sequences**** |
| --- | --- | --- | --- | --- | --- |
| **Target genes** | | | | | |
| 1 | Phenylalanine ammonia lyase | *AsPAL* | MH507022 | A0A481SU87 | F: 5’- GACAACCCGCTCATTGACGT -3’  R: 5’- ATCTCAGCACCCTTGAAGCC -3’ |
| 2 | 4-Coumarate-CoA ligase | *AS4CL* | MH397063 | A0A4Y5UJ50 | F: 5’- CAGAGGCCACAAAGAACACG -3’  R: 5’- GCCGGAGGAACTTGGAATCC-3’ |
| 3 | Caffeoyl-CoA O-methyltransferase | *AsCCoAOMT* | MK577959 | QED40343  AsCCOAOMT  Q7XXP4 | F: 5’- CATGGAGATCGGCGTGTACA -3’  R: 5’- TAGTTGTCGCGGTTGATGTC -3’ |
| 4 | HHT 3* | *AsHHT3* | AB076982 | *AsHHT3*  Q7XXP1 | F: 5’- GTTACTTCGGGAACGTCATCTT -3’  R: 5’- AGTACTCGTCGTCCATCTTCT -3’ |
| 5 | HHT 4 * | *AsHHT4* | MH397064 | A0A4Y5UJ70 | F: 5’- GGTTACTTCGGAAACGTCATCT -3’  R: 5’- AGTAGTCGTCGGACATCCTATC -3’ |
| 6 | HHT 5* | *AsHHT5* | MH397065 | A0A4Y5UJ66 | F: 5’- GGGCGCTGTGGCAGATGGAG -3’  R: 5’- TCCCCAACCGAAATCCGCGTT -3’ |
| 7 | HHT 6* | *AsHHT6* | MH397066 | A0A4Y5UJ73 | F: 5’- GGCATGTCTGGACTTCACTTTA -3’  R: 5’- GTAAGATGGAGTTGGTGGATCG -3’ |
| 8 | Pathogenesis- related protein 5 | *AsPR5* | L39774 | P50695 | F: 5’- GAAGCAGTCGTCCAACATCA-3’  R: 5’- TGTCAACTACAACGGCTGGA-3’ |
| **Housekeeping genes** | | | | | |
| 9 | ADP-ribosylation factor | ADPR | AB050957 |  | F: 5’- CTAGAGATGAGCTCCACAGAATG -3’  R: 5’- CAGCAGCATTCATGGCATTAG -3’ |
| 10 | Glyceraldehyde-3-phosphate dehydrogenase | *GAPDH* | KR029492 |  | F: 5’-GTTTGGCATCGTTGAGGGTT-3’  R: 5’-TGCTGCTGGGAATGATGTTG-3’ |

*HHT: Hydroxycinnamoyl-CoA:hydroxyanthranilate N-hydroxycinnamoyl transferase.

**Primer sequences (1-3 and 6) were taken from Kim *et al*. 2021a. Primer sets 4, 5, 7 and 9 were designed using Primer-BLAST available on NIH (https://www.ncbi.nlm.nih.gov/tools/primer-blast/). Primer sets 8 and 10 were taken from Bahraminejad, 2007 and Tajti *et al*. 2021, respectively.
